# Accelerated hand bone mineral density loss is associated with progressive joint damage in hands and feet in recent-onset rheumatoid arthritis

**DOI:** 10.1186/ar3025

**Published:** 2010-05-20

**Authors:** Melek Güler-Yüksel, Naomi B Klarenbeek, Yvonne PM Goekoop-Ruiterman, Jeska K de Vries-Bouwstra, Sjoerd M van der Kooij, Andreas H Gerards, H Karel Ronday, Tom WJ Huizinga, Ben AC Dijkmans, Cornelia F Allaart, Willem F Lems

**Affiliations:** 1 Department of Rheumatology, Leiden University Medical Center, Albinusdreef 2, 2333 ZA, Leiden, The Netherlands; 2 Department of Rheumatology, VU Medical Center, De Boelelaan 1109, 1007 MB Amsterdam, The Netherlands; 3 Department of Rheumatology, Vlietland Hospital, Burgemeester Knappertlaan 25, 3116 BA Schiedam, The Netherlands; 4 Haga Hospital, Leyweg 275, 2545 CH The Hague, The Netherlands; 5 Department of Rheumatology, Jan van Breemen Institute, Dr Jan van Breemenstraat 2, 1056 AB Amsterdam, The Netherlands

## Abstract

**Introduction:**

To investigate whether accelerated hand bone mineral density (BMD) loss is associated with progressive joint damage in hands and feet in the first year of rheumatoid arthritis (RA) and whether it is an independent predictor of subsequent progressive total joint damage after 4 years.

**Methods:**

In 256 recent-onset RA patients, baseline and 1-year hand BMD was measured in metacarpals 2-4 by digital X-ray radiogrammetry. Joint damage in hands and feet were scored in random order according to the Sharp-van der Heijde method at baseline and yearly up to 4 years.

**Results:**

68% of the patients had accelerated hand BMD loss (>-0.003 g/cm^2^) in the first year of RA. Hand BMD loss was associated with progressive joint damage after 1 year both in hands and feet with odds ratios (OR) (95% confidence intervals [CI]) of 5.3 (1.3-20.9) and 3.1 (1.0-9.7). In univariate analysis, hand BMD loss in the first year was a predictor of subsequent progressive total joint damage after 4 years with an OR (95% CI) of 3.1 (1.3-7.6). Multivariate analysis showed that only progressive joint damage in the first year and anti-citrullinated protein antibody positivity were independent predictors of long-term progressive joint damage.

**Conclusions:**

In the first year of RA, accelerated hand BMD loss is associated with progressive joint damage in both hands and feet. Hand BMD loss in the first year of recent-onset RA predicts subsequent progressive total joint damage, however not independent of progressive joint damage in the first year.

## Introduction

Bone damage in rheumatoid arthritis (RA) includes joint damage and accelerated bone mineral density (BMD) loss [1]. Joint damage is provoked by an increased osteoclast and decreased osteoblast activation, leading to erosive damage, and by proteolytic pathways, leading to cartilage degradation. This is all mostly regulated by TNF-α, IL-1, IL-6, IL-17 and receptor activator of nuclear factor kappa B ligand (RANKL) [2-4]. It is believed that BMD loss, both localized and generalized, is also primarily the effect of increased osteoclast activity in RA [5]. In particular, bones in the proximity of inflamed joints are susceptible to BMD loss due to inflammation [6]. Furthermore, localized hand BMD loss occurs in an early phase of RA [7] and even in pre-RA undifferentiated arthritis [8], and might precede erosive damage on X-ray [9,10].

Dual energy X-ray absorptiometry (DEXA) is the gold standard for measuring BMD. Digital X-ray radiogrammetry (DXR) was developed as a method of radiogrammetry to estimate BMD in the metacarpals using standard hand radiographs [11]. BMD measured by DXR is highly correlated with DEXA measurements and DXR has a high precision for detecting changes in BMD [11,12]. Various clinical studies showed the association between hand BMD loss measured by DXR and RA severity, including disease activity, functional impairment and joint destruction [6,13-22]. Two clinical studies, one of them a pilot study, showed the potential value of BMD loss in hands measured by DXR to predict radiographic joint damage in hands [23,24]. However, to date, no data are available on the association between hand BMD loss and progressive joint damage in hands and feet and on the value of hand BMD loss as predictor of joint destruction in recent-onset RA patients who are treated intensively with disease modifying anti-rheumatic drugs (DMARDs) and TNF-α inhibitors in a tight control setting. We examined the association between accelerated hand BMD loss and progressive joint damage in hands and feet during the first year of recent-onset active RA to see whether both types of bone damage have common pathways in their pathogenesis, and we investigated whether accelerated hand BMD loss in the first year of RA was an independent predictor of subsequent progressive joint damage after four years in patients who are treated in a tight control setting.

## Materials and methods

### Patients

All measures were performed in the setting of the Behandel Strategieën (BeSt) study [25]. Patients aged 18 years and older, who met the definition of RA as defined by the American College of Rheumatology (ACR) 1987 revised criteria, with symptom duration of less than two years and active disease with 6 or more of 66 swollen joints and 6 or more of 68 tender joints and either an erythrocyte sedimentation rate (ESR) of 28 mm/hour or more or a visual analogue scale (VAS) global health of 20 mm or more, and who were DMARD naïve, were included in the trial from April 2000 to August 2002. Exclusion criteria have been reported previously [25]. Of the 508 patients, 236 were excluded from this study predominantly due to switch from analogue to digital radiographs. The other 272 patients had analogue radiographs at both baseline and after one year and were eligible for this study. The baseline and/or one year follow-up analogue radiographs of 16 patients could not be analysed by DXR due to underexposed images (13 patients) or improper positioning of the hands (3 patients). Hence, 256 patients were included in the current study.

### Study design

The BeSt study was conducted by rheumatologists participating in the Foundation for Applied Rheumatology Research, in 18 peripheral and 2 university hospitals in the western part of the Netherlands. The medical ethics committee at each participating center approved the study protocol and all patients gave written informed consent prior to participation in the study.

After inclusion, patients were randomized to be treated according to one of four treatment strategies: sequential monotherapy starting with methotrexate (MTX); step-up combination therapy also starting with MTX; initial combination therapy with quickly tapered high-dose prednisone, MTX and sulphasalazine, or initial combination therapy with infliximab and MTX. For all groups, the protocol described a number of subsequent treatment steps for patients whose response to therapy was insufficient, based on the disease activity score (DAS) in 44 joints of more than 2.4. The treatment protocol and the effect of the different treatment strategies on hand BMD loss after one and two years are described earlier in detail [6,25].

Concomitant treatment with non-steroidal anti-inflammatory drugs and intra-articular corticosteroids were permitted but not parenteral corticosteroids. In case of calcium intake of less than 1,000 mg/day and serum vitamin D level below the local reference value at baseline, suppletion of 500 to 1,000 mg/day calcium and 400 IE/day vitamin D (colecalciferol), respectively, was advised. Antiresorptive therapy with oral alendronate, 10 mg/day or 70 mg/week, or risedronate, 5 mg/day or 35 mg/week, was advised if the BMD measurement at baseline showed at a T-score -2.5 standard deviations (SD) or less in the spine and/or hip in non-corticosteroid users or a T-score -1 SD or less in corticosteroids users (ACR recommendations) [6].

### Hand BMD measurements

Analogue radiographs of both hands in the posterior-anterior view were digitized by a high-resolution 300 DPI scanner (Canon Vidar VXR-12 plus, Amstelveen, North-Holland, The Netherlands) and analysed under blind conditions using the DXR-online (Pronosco X-posure system, Sectra, Sweden). According to the manufacturer, there is a very good agreement between BMD measured by DXR on original analogue radiographs and on digitalized versions. Patients who switched from analogue to digital radiographs were excluded due to lack of comparability between the different imaging devices.

DXR is a computerized version of the traditional technique of radiogrammetry originally proposed by Barnett and Nordin [26]. The digitized hand radiograph is subjected to a number of image processing algorithms to measure the cortical thickness of three regions of interest around the narrowest part of the second, third and fourth metacarpal bones [11]. A mean surrogate BMD, based on the mean volume per area, was calculated in g/cm^2 ^with correction for the estimated porosity. Both hands were measured and the mean was used for the analyses.

Hand BMD loss after one year was categorized in two groups using the cut-off of -0.003 g/cm^2^/year, equal to the upper limit of normal BMD loss in the metacarpals according to specifications by the manufacturer.

### Radiographic assessment of progressive joint damage

Radiographic progression of joint damage in hands and feet at baseline and after one to four years were independently scored by two readers, blinded for patient characteristics, treatment group and time order, using the Sharp-van der Heijde score (SHS) [27]. The inter-class correlation coefficient between the readers was 0.98. The mean score of the two observers were used for the analyses.

Progressive joint damage in the first year was defined as an increase in total SHS of 5 units or more at year one compared with baseline. In subanalyses, progressive joint damage in hands was defined as delta SHS 0 to 1 year 5 units or more, whereas progressive joint damage in feet was defined as delta SHS 0 to 1 year 3 units or more, due to a 0.6 times lower maximum score in feet than in hands. Subsequent progressive total joint damage in hands and feet was defined as delta SHS 1 to 4 years 5 units or more.

### Demographic and clinical variables

The following variables were collected at baseline: age; sex; and symptom duration. At baseline and after one year the following variables were collected: postmenopausal status; body mass index (BMI); DAS; based on the number of swollen joints and the Ritchie articular index (RAI) for pain in tender joints; the VAS for patient's global assessment of disease activity (0 to 100 mm); ESR; C-reactive protein (CRP); serum IgM rheumatoid factor (RF), defined as positive or negative according to locally applied assays and cut-off units; and functional disability by the Dutch validated health assessment questionnaire (HAQ). The presence of anti-citrullinated protein antibodies (ACPA) was determined from serum samples obtained at baseline or during follow up. The presence or absence of ACPA is a stable characteristic [28].

### Statistical analyses

All analyses were performed in an intention-to-treat method using all available data.

To determine the associations between hand BMD loss and progressive joint damage in hands and feet after one year, multivariate logistic regression analyses were performed adjusted for age, gender, postmenopausal status, BMI, HAQ, baseline SHS, treatment group, and the use of intra-articular steroids and antiresorptive drugs (bisphosphonates, vitamin D and calcium supplements and hormone replacement therapy (HRT)).

The sensitivity, specificity, and positive and negative predictive value of hand BMD loss with regard to total progressive joint damage in the first year were calculated. Various baseline demographic and disease-related factors and one-year follow-up disease-related factors were analysed regarding prediction of subsequent progressive total joint damage after four years by univariate logistic regression analyses adjusted for age, gender, postmenopausal status, BMI and HAQ, and additionally adjusted for the treatment group and use of antiresorptive drugs and intra-articular steroids during the first year follow-up in case of one-year follow up variables. The following factors were analysed: baseline demographic factors (gender, age ≥ 50 years, postmenopausal status and BMI ≥ 25 kg/m^2^), baseline disease-related factors (symptom duration ≥ 6 months, presence ACPA and RF, number of swollen joints ≥ 10, RAI ≥ 10, ESR ≥ 30 mm/hr, CRP ≥ 10 mg/L, HAQ ≥ 1.057 units [29] and SHS ≥ 1 unit) and one-year follow-up disease-related factors (high area under the curve (AUC) of number of swollen joints, RAI, ESR and CRP and delta HAQ ≤ -0.22 units [30], total SHS ≥ 5 units and hand BMD loss >-0.003 g/cm^2^). Both significant (p-value < 0.05) and borderline significant (0.05<*P *< 0.10) predictors derived by these univariate analyses were entered in multiple multivariate logistic regression analyses to determine the independent predictors of subsequent progressive joint damage.

## Results

### Patient characteristics

The baseline characteristics of the 256 patients included in the study and 252 patients excluded are shown in Table 1. Patients included had shorter disease duration, were less frequently ACPA positive and had less damage in the feet, especially less cartilage degradation, compared with the non-included patients. With regard to randomization into the four treatment groups by age, sex, RF, DAS, ESR level, CRP level, HAQ score, hand SHS and BMD (obtained in 107 patients at baseline who were excluded from this study), there were no significant differences between patients who were enrolled in this study and who were not.

**Table 1:** Baseline demographic and disease characteristics from patients from the BeSt cohort who are included and not included in this study

Demographic variables	Patients included in study (n = 256)	Patients not included in study (n = 252)	*P *value
Age, years^†^	54 (14)	54 (13)	0.941
Women, %	65	70	0.208
Postmenopausal, %	66	68	0.817
Randomization between the treatment groups, %	Sequential monotherapy: 25 Step-up therapy: 23 Initial combi therapy with prednisone: 27 Initial combi therapy with infliximab: 26	Sequential monotherapy: 25 Step-up therapy: 25 Initial combi therapy with prednisone: 25 Initial combi therapy with infliximab: 25	0.921
**Disease related variables**			

Symptom duration, weeks^‡^	24 (14-53)	23 (13-53)	0.929
Disease duration, weeks^‡^	2 (1-5)	3 (1-5)	0.011
ACPA positive, %, n = 247 (not all baseline)	54	70	0.000
RF positive, %	62	68	0.175
DAS^†^	4.4 (0.9)	4.4 (0.9)	0.529
ESR^‡^	37 (19-54)	36 (19-57)	0.781
CRP^‡^	20 (9-58)	26 (10-55)	0.272
HAQ score, 0-3 scale^†^	1.4 (0.6)	1.4 (0.7)	0.276
			
Total SHS, 0-448 scale^‡, † ^n = 248	5.9 (8.2)/2.5 (0.5-8.5)	8.7 (12.7)/4.3 (1.0-11.0)	0.024*
Erosion score, 0-280 scale^‡, †^	2.8 (4.7)/1 (0.0-3.5)	3.9 (6.2)/1.5 (0.0-5.0)	0.011*
JSN score, 0-168 scale^‡, †^	3.0 (4.8)/1.0 (0.0-4.1)	4.8 (7.7)/2.0 (0.0-5.6)	0.041*
			
Total SHS hands, 0-280 scale^‡, †^	3.0 (4.8)/1.0 (0.0-3.5)	4.6 (8.4)/1.0 (0.0-5.0)	0.217*
Erosion score hands, 0-160 scale^‡, †^	0.9 (1.8)/0.0 (0.0-1.0)	1.4 (3.1)/0.5 (0.0-1.0)	0.112*
JSN score hands, 0-120 scale^‡, †^	2.1 (3.9)/0.0 (0.0-3.0)	3.1 (6.0)/0.50 (0.0-3.6)	0.247*
			
Total SHS feet, 0-168 scale^‡, †^	2.8 (5.4)/0.5 (0.0-3.0)	4.1 (7.2)/1.5 (0.0-4.5)	0.011*
Erosion score feet, 0-120 scale^‡, †^	1.9 (3.9)/0.5 (0.0-2.0)	2.5 (4.6)/0.5 (0.0-2.5)	0.123*
JSN score feet, 0-48 scale^‡, †^	0.9 (2.0)/0.0 (0.0-1.0)	1.7 (3.4)/0.0 (0.0-2.0)	0.013*
Presence erosive damage ≥ 1 unit,	70	74	0.321
% n = 248	28	34	0.166
Presence erosive damage hands ≥ 1 unit, %	40	46	0.205
Presence erosive damage feet ≥ 1 unit, %			
Hand BMD (g/cm^2^)^†^	0.59 (0.08)	0.59 (0.09) n = 107	0.870

†Mean (standard deviation); ‡ median (interquartile range); * *P *values derived from non-parametric tests.

ACPA, anti-citrullinated protein antibodies; BMI, body mass index; CRP, C-reactive protein; DAS, disease activity score; ESR, erythrocyte sedimentation rate; HAQ, health assessment questionnaire; JSN, joint space narrowing; RF, rheumatoid factor; SHS, Sharp-van der Heijde score.

Of the study population, 65% were females, 66% of them postmenopausal, and the mean age was 54 years. At baseline the patients had a median symptom duration of 24 weeks and mean (SD) DAS of 4.4 (0.9). RF was positive in 62% of the patients and 70% had at least one erosion in hands and feet.

### Changes in hand BMD and joint damage in hands and feet in the first year

The median (interquartile range (IQR)) hand BMD change was, in absolute value, -0.0088 g/cm^2 ^(-0.021 to -0.0005) and in percentage of baseline BMD -1.4% (-3.8% to -0.1%) after one year. On the individual level, 68% of patients had accelerated hand BMD loss of more than -0.003 g/cm^2^, from now on called hand BMD loss. The mean (SD) progression of total SHS in hands and feet, and SHS in hands and feet separately was 3.0 (11.3), 1.9 (7.0) and 1.1 (5.0), respectively. After one year 18%, 12% and 11% of the patients had progressive total joint damage of 5 units or more, hand joint damage 5 units or more and feet joint damage of 3 units or more, respectively.

**Figure 1:**
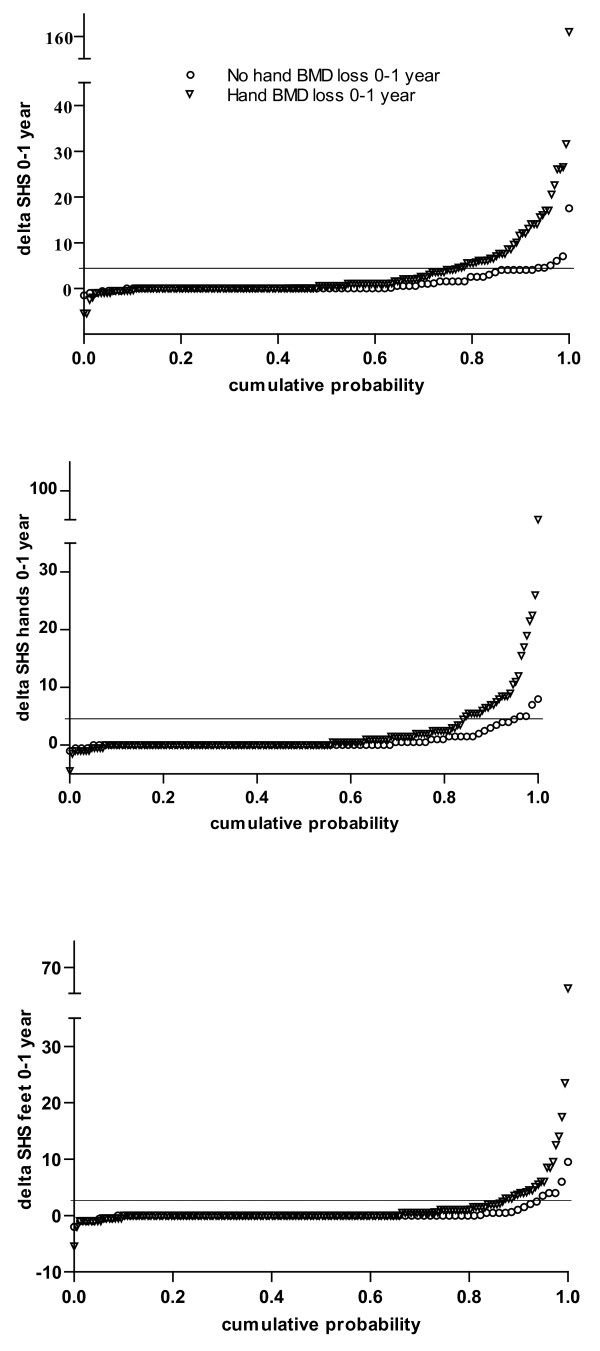
**Cumulative probability plot of changes in Sharp-van der Heijde score (SHS)**. Results are shown in both hands and feet, in only hands and in only feet after one year in recent-onset active rheumatoid arthritis patients with accelerated hand bone mineral density (BMD) loss (triangles) and without accelerated hand BMD loss (circles) after one year.

In patients with hand BMD loss the mean (SD) progression of total SHS after one year was 4.0 (13.6) compared with 1.1 (2.6) in patients without hand BMD loss (*P *= 0.036 derived by non-parametric test). Hand BMD loss after one year was significantly associated with higher progression rates both in hands (2.5 (8.4) versus 0.8 (1.7), *P *= 0.033) and feet (1.4 (5.9) versus 0.4 (1.5), *P *= 0.047). The cumulative probability plots of changes in total SHS and changes in hands and feet SHS separately after one year in patients with and without hand BMD loss are shown in Figure 1. Multivariate logistic regression analyses adjusted for possible confounders were performed to study the independent associations between hand BMD loss and progressive total joint damage in hands and feet. Progressive total joint damage in hands and feet after one year was independently associated with hand BMD loss with an odds ratio (OR) (95% confidence interval (CI)) of 10.6 (2.6 to 42.7; *P *= 0.001). In separate analyses, hand BMD loss was associated with progressive joint damage in both hands and feet after one year, although more strongly in hands (OR (95% CI) 5.3 (1.3 to 20.9)) than in feet (3.1 (1.0 to 9.7)). Both erosion and joint space narrowing (JSN) score in hands and feet contributed equally to the association with hand BMD loss (data not shown).

### Sensitivity, specificity and predictive value of hand BMD loss in the first year

The sensitivity of hand BMD loss for detecting progressive total joint damage after one year was 39 of 45 (87%) and the specificity 74 of 203 (36%). The positive predictive value, the probability of the presence of progressive joint damage when hand BMD loss is present, was 39 of 168 (23%), whereas the negative predictive value, the probability of absence of progressive joint damage when hand BMD loss is absent, was 74 of 80 (93%).

### Predictors of subsequent progressive radiographic damage after four years

**Figure 2:**
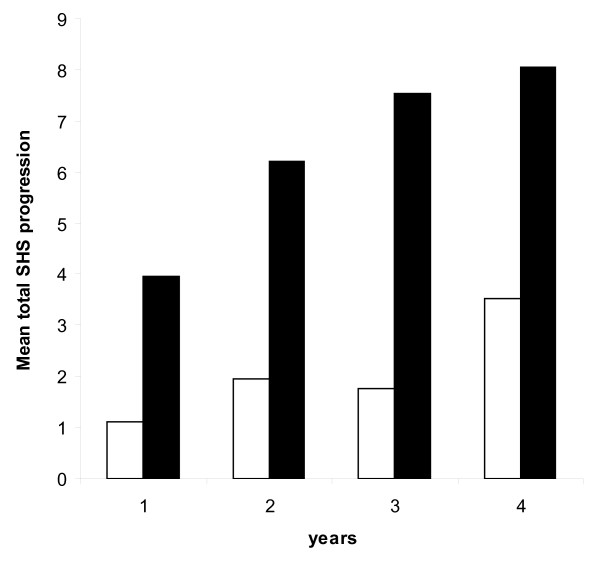
**Mean progression of total Sharp-van der Heijde (SHS) in hands and feet after up to four years in patients with (black columns) and without (white columns) hand bone mineral density (BMD) loss in the first year of rheumatoid arthritis**. The differences in mean total SHS progression after one, two, three and four years between patients with and without hand BMD loss in the first year are all significant (*P *< 0.05 derived by non-parametric tests).

The mean (SD) cumulative progression of total SHS in hands and feet was 3.0 (11.3), 4.9 (14.5), 5.8 (16.7) and 6.6 (13.3) after one to four years compared with baseline. After one to four years, 18%, 26%, 27% and 30% of the patients, respectively, had progressive total joint damage of 5 units or more. The association between hand BMD loss in the first year and progressive total joint damage remained over time up to four years (Figure 2).

The mean (SD) progression of total SHS was 2.9 (7.6) after four years compared with year one and 14% of the patients had progressive total joint damage of 5 units or more after four years compared with year one. To investigate whether hand BMD loss in the first year could predict long-term damage progression, univariate logistic regression analyses were performed with subsequent progressive total joint damage after four years of 5 units or more compared with year one as dependent variable and various potential baseline and one year follow-up predictors as independent variables adjusted for possible confounders (Table 2). Of the baseline variables, the presence of ACPA, RF and joint damage at baseline were significant predictors of subsequent progressive total joint damage after four years. Of the one-year follow-up variables, a high AUC of ESR and CRP, progressive total joint damage of 5 units or more and hand BMD loss were significant predictors of subsequent progressive joint damage after four years. The association of hand BMD loss with subsequent progressive joint damage was less strong (OR (95% CI) 3.1 (1.3 to 7.6)) than the association with progressive joint damage in the first year (OR (95% CI) 30.7 (9.4 to 100)).

**Table 2:** Baseline and one-year follow-up predictors of subsequent progressive total joint damage in hands and feet after four years derived from univariate logistic regression analyses

	**Progressive total joint damage 1-4 ≥ 5 units**		
	
	**β**	**OR (95% CI)**	***P *value**
	
**Baseline variables**			
Female gender	-0.28	0.76 (0.38-1.49)	0.418
Age ≥ 50 years	-0.11	0.90 (0.47-1.71)	0.744
Postmenopausal status	0.56	1.75 (0.70-4.40)	0.232
BMI ≥ 25 kg/m^2^	-0.54	0.58 (0.31-1.11)	0.100
Symptom duration ≥ 6 months	-0.02	0.98 (0.52-1.87)	0.958
Presence ACPA	1.57	4.80 (1.39-16.6)	0.001
Presence RF	1.14	3.11 (1.43-6.78)	0.004
Number of swollen joints ≥ 10	-0.42	0.66 (0.32-1.33)	0.243
Ritchie articular index ≥ 10	-0.39	0.68(0.34-1.36)	0.276
ESR ≥ 30 mm/hr	0.12	1.13 (0.59-2.18)	0.714
CRP ≥ 10 mg/L	0.63	1.88 (0.82-4.31)	0.136
HAQ ≥ 1.057 units	-0.39	0.67 (0.35-1.30)	0.240
SHS ≥ 1 unit	1.99	7.29 (1.70-31.14)	0.007
			
**First year follow-up variables**			
High AUC number of swollen joints	-0.002	0.998 (0.991-1.006)	0.692
High AUC Ritchie articular index	-0.003	0.997 (0.992-1.003)	0.325
High AUC ESR	0.002	1.002 (1.001-1.004)	0.003
High AUC CRP	0.003	1.003 (1.001-1.004)	0.000
Delta HAQ ≤ -0.22 units	0.273	1.31 (0.56-3.09)	0.531
Progressive SHS ≥ 5 units	3.42	30.7 (9.4-100.1)	0.000
Hand BMD loss >0.003 g/cm^2^	1.15	3.14 (1.30-7.57)	0.011

All variables are adjusted for age, gender, postmenopausal status, BMI and HAQ. First follow-up variables are additionally adjusted for treatment group and the use of intraarticular corticosteroids injections and anti-resorptive therapy (bisphosphonates, calcium and vitamin D suppletion and hormone replacement therapy).

ACPA, anti-citrullinated protein antibodies; AUC, area under the curve; BMD, bone mineral density; BMI, body mass index; CI, confidence interval; CRP, C-reactive protein; ESR, erythrocyte sedimentation rate; HAQ, health assessment questionnaire; OR, odds ratio; RF, rheumatoid factor; SHS, Sharp-van der Heijde score.

Multiple multivariate regression analyses were performed to investigate the predictive ability of different factors and the mutual interaction between them. In the first multivariate model, all (borderline) significant predictors from the univariate analyses were entered, adjusted for possible confounders (Table 3). The presence of ACPA was an independent predictor of progressive joint damage with an OR (95% CI) of 3.1 (1.4 to 6.1). Progressive joint damage in the first year was a strong and independent predictor of subsequent progressive joint damage with an OR (95% CI) of 27.1 (10.9 to 67.4). Hand BMD loss in the first year with the diagnosis of RA was not an independent predictor anymore (*P *= 0.688). The adjusted R^2^, estimating the proportion of variance in progressive joint damage that is explained by the predictors, was 0.53.

**Table 3:** Baseline and one-year follow-up predictors of subsequent progressive total joint damage in hands and feet after four years derived from multivariate logistic regression analysis

	**Progressive total joint damage 1-4 ≥ 5 units**		
	
	**β**	**OR (95% CI)**	***P *value**
	
**Baseline variables**			
Presence ACPA	1.20	3.14 (1.37-6.12)	0.015
Presence RF	0.59	1.80 (0.55-6.08)	0.314
SHS ≥ 1 unit	0.85	2.55 (0.48-13.0)	0.342
			
**First year follow-up variables**			
High AUC ESR	0.001	1.001 (0.998-1.004)	0.444
High AUC CRP	0.001	1.001 (0.999-1.004)	0.339
Progressive SHS ≥ 5 units	3.35	27.1 (10.9-67.4)	0.000
Hand BMD loss >0.003 g/cm^2^	0.30	1.30 (0.38-3.84)	0.688
R^2^, adjusted		0.534	

All variables with a *P *< 0.10 in the univariate analyses were entered in this multivariate analysis corrected for age, gender, postmenopausal status, BMI and health assessment questionnaire and first year follow-up variables additionally corrected for the use of anti-resorptive therapy (bisphosphonates, calcium and vitamin D suppletion and hormone replacement therapy) and intraarticular corticosteroid injections during first year and treatment group during.

ACPA, anti-citrullinated protein antibodies; AUC, area under the curve; BMD, bone mineral density; BMI, body mass index; CI, confidence interval; CRP, C-reactive protein; ESR, erythrocyte sedimentation rate; OR, odds ratio; RF, rheumatoid factor; SHS, Sharp-van der Heijde score.

In the second multivariate model all (borderline) significant predictors from the univariate analyses were entered, except progressive joint damage in the first year, adjusted for possible confounders (Table 4). Hand BMD loss in the first year was a predictor of subsequent progressive joint damage independent of the presence of auto-antibodies, joint damage at baseline and high AUC of ESR and CRP with an OR (95% CI) of 3.0 (1.1 to 8.8). The adjusted R^2 ^was considerably lower at 0.29.

**Table 4:** Baseline and one-year follow-up predictors, progressive SHS of 5 units or more in the first year excluded, of subsequent progressive total joint damage in hands and feet after four years derived from multivariate logistic regression analysis

	**Progressive joint damage 1-4 ≥ 5 units**		
	
	**β**	**OR (95% CI)**	***P *value**
	
**Baseline variables**			
Presence ACPA	1.30	3.95 (1.17-15.0)	0.017
Presence RF	0.11	1.10 (0.38-2.98)	0.803
SHS ≥ 1 unit	1.81	5.78 (1.23-28.1)	0.020
			
**First year follow-up variables**			
High AUC ESR	0.002	1.002 (0.999-1.004)	0.160
High AUC CRP	0.002	1.002 (1.000-1.004)	0.059
Progressive SHS ≥ 5 units	-	-	-
Hand BMD loss >0.003 g/cm^2^	1.10	3.00 (1.12-8.81)	0.035
R^2^, adjusted		0.290	

All variables with a *P *< 0.10 in the univariate analyses, except progression SHS of 5 units or more in the first year, were entered in this multivariate analysis corrected for age, gender, postmenopausal status, BMI and health assessment questionnaire and first year follow-up variables additionally corrected for the use of anti-resorptive therapy (bisphosphonates, calcium and vitamin D suppletion and hormone replacement therapy) and intraarticular corticosteroid injections during first year and treatment group during.

ACPA, anti-citrullinated protein antibodies; AUC, area under the curve; BMD, bone mineral density; BMI, body mass index; CI, confidence interval; CRP, C-reactive protein; ESR, erythrocyte sedimentation rate; OR, odds ratio; RF, rheumatoid factor; SHS, Sharp-van der Heijde score.

To explore further the usefulness of progressive joint damage in the first year as a predictor of subsequent progressive damage, joint damage progression in the first year was divided in to four groups: no progression (SHS ≤ 0 unit, the reference group), dubious progression (0<SHS<5 units), moderate progression (5 ≤ SHS<10 units) and high progression (SHS ≥ 10 units). They were then entered in a third multivariate regression analysis together with all (borderline) significant predictors from the univariate analyses and possible confounders. This analysis showed that even dubious progressive joint damage was an independent predictor of subsequent progressive joint damage with an OR (95% CI) of 5.5 (1.3 to 24) and that the ORs (95% CIs) were considerably higher when the progressive joint damage in the first year was moderate, 68 (14 to 345), or high, 144 (20 to 1045).

## Discussion

This study into the association between hand BMD loss and radiographic joint damage progression shows that in the first year of RA hand BMD loss is associated with progressive joint damage in hands and feet, and that the association seems stronger with damage in hands than in feet. Moreover, hand BMD loss in the first year predicts subsequent progressive total joint damage: however, not independent of progressive joint damage in the first year.

The relation between BMD loss and progressive joint damage in the first year of RA suggests that both types of bone damage share common pathways in their pathogenesis and are the result of the same inflammatory process. It is thought that BMD loss in RA patients is caused, just like joint damage, by increased osteoclast activation, mainly regulated by TNF-α, IL-1, IL-6, IL-17 and RANKL [2,3]. This is also in line with *in vitro *studies showing increased osteoclast functional activity in RA patients with generalized osteopenia [5]. A stronger association between hand BMD loss and progressive joint damage in hands compared with damage in feet also suggests that bones in the direct proximity of the inflammatory activity are more susceptible to BMD loss due to, besides the systemic, the local effect of high pro-inflammatory cytokine levels originating in adjacent active arthritis of the hand joints. On the other hand, it may also be partially explained by methodological issues. Firstly, less joint damage in feet can be detected due to less evaluated joints in feet than in hands. To limit this problem, we used a lower cut-off point to define progressive damage in feet (3 units in feet versus 5 units in hands due to 0.6 times lower maximum score in feet) and after this correction the percentages of patients having progressive joint damage in the hands and in feet were similar. Secondly, the patients in this subanalysis had significant less damage in the feet than the patients who were excluded from this subanalysis; however, in absolute terms, our population had active disease with high DAS and erosions present in the majority of patients at baseline.

In the first year of RA, hand BMD loss was seen in 68% of the patients, whereas progressive joint damage was seen in only 18%. There are several explanations for this disassociation. First, localized BMD loss occurs mostly earlier in and more often during the disease course than advanced joint damage to bone and cartilage, especially in recent-onset RA [9,10]. This is emphasized by the sensitivity, specificity and predictive value of hand BMD loss with regard to progressive joint damage in the first year. Both the sensitivity and negative predictive value were high, 87% and 93%, respectively, whereas the specificity and positive predictive value were low, 36% and 23%, respectively, suggesting that most patients with progressive joint damage also have hand BMD loss at the same time, whereas in most patients with hand BMD loss progressive joint damage is absent. A second explanation might be that the technique of measurement of BMD loss by DXR is more sensitive to detect significant changes in cortical BMD during a follow-up period, while progressive joint damage as measured by the semi-objective SHS method is less sensitive to detect significant changes in structural damage, both erosions and JSN, during the same follow-up period.

We showed that hand BMD loss in the first year of RA is a predictor of subsequent progressive total joint damage, independent of the presence of auto-antibodies and joint damage at baseline. This is in accordance with the findings of Hoff and colleagues, who in RA patients with mean disease duration of 2.2 years at inclusion also showed that hand BMD loss was a predictor of progressive damage in hands after 5 and 10 years, independent of baseline predictors, such as joint damage at baseline and the presence of ACPA [24]. However, hand BMD loss is probably predicting progressive joint damage because hand BMD loss itself incorporates the effect of inflammation over time, as opposed to other factors that are static measures of the situation at baseline. Therefore we compared the predictive value of hand BMD loss with changes in other potential one-year follow-up predictors in multivariate regression analyses, and found that radiographic progressive joint damage is a much stronger predictor for subsequent progressive damage and that hand BMD loss was not predicting subsequent progressive damage independent of progressive damage in the first year probably due to the common inflammatory pathway between BMD loss and joint damage.

As progressive joint damage in the first year is superior as a predictor of further joint damage progression, in daily practice hand BMD loss after one year will not add to the identification of patients at risk for further destruction in recent-onset active RA. However, as hand BMD measurements by DXR are highly precise in detecting changes [12], early BMD evaluation, at three to four months after disease onset or even in the undifferentiated stage of the disease, might be a useful tool to predict poor outcome in these patients.

It might be argued whether progression of SHS is useful in clinical practice as a predictor, because it is a complicated scoring method that requires special training to perform. To mimic the daily clinical practice of radiographic assessment, we categorized the progression of joint damage in four categories: patients with no, dubious, moderate or high progression. We found that even patients with dubious progressive damage in the first year had 5-fold more subsequent progressive damage, and with moderate and high progression even 65-fold and 138-fold more than patients with no progression, while hand BMD loss was associated with 3-fold more subsequent damage. Furthermore hand BMD loss measured by the DXR technology also requires special equipment, in general not available in medical centers, or payments for the measurements when the online service is used.

Further the fact that there are significant differences in baseline variables between patients who were included in this trial and patients who were not included might be argued. The included patients have shorter disease duration, are less often ACPA positive and have less joint damage at baseline, suggesting that patients with relatively less active disease were included in this trial. Nevertheless, in absolute terms, the included patients had high disease activity with erosive damage in the majority, even in this early stage of the disease.

## Conclusions

In the first year of RA, accelerated hand BMD loss is associated with progressive joint damage in both hands and feet. Hand BMD loss in the first year is a predictor of subsequent progressive total joint damage; however, it is not independent of progressive joint damage in the first year, which remains the strongest predictor of subsequent damage. These findings suggest that both methods detect effects in a common pathway of osteoclastic activity and that initial joint damage progression in the first year of RA is superior in predicting later progressive joint damage.

## Abbreviations

ACPA: anti-citrullinated protein antibodies; ACR: American College of Rheumatology; AUC: area under the curve; BMD: bone mineral density; BMI: body mass index; CI: confidence interval; CRP: C-reactive protein; DAS: disease activity score; DEXA: dual energy X-ray absorptiometry; DMARDs: disease modifying anti-rheumatic drugs; DXR: digital X-ray radiogrammetry; ESR: erythrocyte sedimentation rate; HAQ: health assessment questionnaire; HRT: hormone replacement therapy; IL: interleukin; IQR: interquartile range; JSN: joint space narrowing; MTX: methotrexate; OR: odds ratio; RA: rheumatoid arthritis; RAI: ritchie articular index; RANKL: receptor activator of nuclear factor kappa B ligand; RF: rheumatoid factor; SD: standard deviation; SHS: Sharp-van der Heijde score; TNF-α: tumor necrosis factor alpha; VAS: visual analogue score.

## Competing interests

CF Allaart received lecture fees from Schering-Plough. BAC Dijkmans has received funds for research and lecture fees from Schering-Plough.

## Authors' contributions

MG-Y, YPMG-R, JKdV-B, WJH, BACD, CFA and WFL contributed to study design. MG-Y, NBK, YPMG-R, JKdV-B, SMvdK, WJH, BACD and CFA contributed to study coordination. MG-Y, NBK, YPMG-R, JKdV-B, SMvdK, AHG, HKR, WJH, BACD, CFA and WFL contributed to acquisition of data. MG-Y contributed to statistical analysis. MG-Y, WJH, BACD, CFA, and WFL contributed to analysis and interpretation of data. MG-Y, WJH, BACD, CFA and WFL contributed to manuscript preparation. All authors read and approved the final manuscript.
